# Incidence of cutaneous melanoma in patients with histologically confirmed dysplastic naevus: A follow‐up study in a large UK Healthcare Trust

**DOI:** 10.1002/ski2.44

**Published:** 2021-05-07

**Authors:** L. McDonald, C. Clarke, V. O’Neill, J. Houghton, O. Dolan, C. McCourt

**Affiliations:** ^1^ Department of Dermatology Royal Victoria Hospital Belfast Health and Social Care Trust Belfast UK; ^2^ Department of Histopathology Royal Victoria Hospital Belfast Health and Social Care Trust Belfast UK; ^3^ School of Medicine, Dentistry and Biomedical Sciences Queen's University Belfast Belfast UK

1

Dear Editor,

Management of dysplastic naevi (DN) is controversial. Opinion is divided regarding their definition, histological grading, clinical significance and risk of subsequently developing cutaneous melanoma (CM). A recent systematic review by Vuong et al.^1^ evaluated the outcomes of observed versus re‐excised histologically confirmed DN, with data largely drawn from North American centres. The recent multicentre study by Kim et al.^2^ helped to further elucidate the risk of subsequent cutaneous melanoma in patients with DN and guide pragmatic management of moderately severe DN, but there remains a lack of overarching consensus guidance for clinicians and a notable paucity of outcome data in the United Kingdom. We sought to contribute to the international debate with a study aiming to evaluate outcomes for patients with histologically proven DN and the incidence of subsequent CM in a large UK centre.

A retrospective single‐centre cohort study was conducted of cases of histologically confirmed DN reported in the Trust Pathology Database using standardised SNOMED codes between 1 January 2008 and 31 December 2013. All reports were manually verified by a clinician. Cases were analysed for demographics, referral source, anatomical site, procedure type, clearance margins, histological severity grading, re‐excision and development of subsequent CM (Table 1).

**TABLE 1:** Characteristics of dysplastic naevi and demographics of patients who developed subsequent separate site CM

Characteristic		CM at separate site		Total		*p* value
		Yes	No			
**Dysplastic naevi (*n* = 248)**				***n* = 248**	**%**	
Anatomical site	Anterior trunk	–	–	45	18.1	
	Back	–	–	96	38.7	
	Groin/buttocks	–	–	5	2.0	
	Head and neck	–	–	6	2.4	
	Legs/feet	–	–	43	17.3	
	Arms/shoulders/hands	–	–	34	13.7	
	Not specified	–	–	19	7.7	
Type of biopsy	Excisional biopsy	–	–	232	93.5	
	Incisional biopsy	–	–	15	6.0	
	Unknown	–	–	1	0.4	
Margin clearance	Yes	–	–	234	94.4	
	No	–	–	13	5.2	
	Not documented	–	–	1	0.4	
Grade of dysplasia	Mild	–	–	3	1.2	
	Moderate	–	–	4	1.6	
	Severe	–	–	6	2.4	
	Not documented	–	–	235	94.8	
Specimen source	Primary care	–	–	19	7.7	
	Secondary care	–	–	229	92.3	

**Unique patients (*n* = 236)**		***n* = 6**	***n* = 230**	***n* = 236**	**%**	
Melanoma	Previous history	3	21	24	10.2	0.0152
	No history	3	209	212	89.8	
Sex	Male	4	99	103	43.6	0.4081
	Female	2	131	133	56.4	
Age	Median	63	43	44	–	0.006
	Range	49‐79	16‐82	16‐82	–	

Abbreviation: CM, cutaneous melanoma.

A total of 248 cases were evaluated in 236 unique patients (103 male, 133 female) with a median (range) age at diagnosis of 44 (16, 82) and median (range) follow‐up period of 75 (60, 131) months. One patient died 2 years before the end of the follow‐up period of an unrelated cause. The majority of specimens (93.55%) were excisional biopsies in keeping with national guidelines^3^ and 92.34% of procedures were performed in Secondary care. The back was the commonest anatomical site (38.7%). Only 13 (5.25%) of cases had grade of dysplasia documented. Histological margins were reported as clear in 234 (94.4%) cases. Of this group, six were re‐excised on the basis of ‘close’ margins with no change in diagnosis. One case had no specified margin clearance. The remaining 13 (5.2%) of cases had documented involved histological margins. Ten of these underwent re‐excision. Two were upgraded, one to melanoma in situ and the other to superficial spreading melanoma. Two cases with involved margins were not re‐excised with no adverse outcome reported within the study period. One patient had clinical recurrence of a fully excised but ungraded DN after 5 years. Subsequent re‐excision was reported as a severely DN with no further adverse outcome reported within the study period. No patient developed a subsequent CM at the site of previous DN biopsy.

Six patients developed a separate site CM (two melanoma in situ and four melanoma). A third of these had a prior personal history of CM. Three patients were diagnosed concurrently with a separate site CM on the same day as the diagnosis of DN. The crude incidence rate of subsequent separate site CM in all patients with histologically confirmed DN was 374.73 per 100 000 person years. Indirect age‐standardisation of our cohort using the Northern Ireland Cancer Registry incidence rates of melanoma for 2014–2018^4^ showed a standardised incidence ratio of 13.32 (95% confidence interval [CI]: 4.86–28.99) for all patients and 7.73 (95% CI: 1.55–22.59) after exclusion of cases with previous history of CM. Age in the group who developed a subsequent separate site CM was statistically significantly higher than in the group who did not (*U* = 238, *p* = 0.006). A prior history of CM was significantly associated with the risk of developing a subsequent separate site CM (odds ratio: 9.95; 95% CI: 1.89–52.45).

This was a retrospective review with inherent limitations. Although a single‐centre study our Healthcare Trust is one of the largest in the United Kingdom with a Pathology Department servicing just under a million people. It provides tertiary dermatology and plastic surgery services, possibly introducing bias in terms of the complexity of cases. We have a small cohort of dedicated reporting Dermatopathologists, however, which may have mitigated against interpathologist reporting variability. Lack of clinical information precluded other risk factor analyses. Finally, the study period predates the introduction of the ‘Tissue pathway for Dermatopathology’ by the Royal College of Pathologists UK^5^ which specifically comments on severity grading.

To our knowledge, this is the first follow‐up study reporting clinical outcomes for histologically proven DN in a UK Centre. Our study supports broad opinion that the risk of transformation of an individual DN to CM is low. However, despite the study limitations, given the increased risk of subsequent separate site CM in patients with DN, we add to the growing body of literature that suggests DN should be considered as a surrogate marker for increased skin surveillance, particularly in those with a prior history of CM.

## CONFLICT OF INTEREST

The author declares that there is no conflict of interest.

## References

[1] Vuong KT , Walker J , Powell HB , Thomas NE , Jonas DE , Adamson AS . Surgical re‐excision vs. observation for histologically dysplastic naevi: a systematic review of associated clinical outcomes. Br J Dermatol. 2018;179:590–8. 29570779 10.1111/bjd.16557

[2] Kim CC , Berry EG , Marchetti MA , Swetter SM , Lim G , Grossman D , et al. Risk of subsequent cutaneous melanoma in moderately dysplastic nevi excisionally biopsied but with positive histologic margins. JAMA Dermatol. 2018;154(12):1401–8. 30304348 10.1001/jamadermatol.2018.3359 PMC6583364

[3] Marsden JR , Newton‐Bishop JA , Burrows L , Cook M , Corrie PG , et al. Revised U.K. Guidelines for the management of cutaneous melanoma 2010. Br J Dermatol. 2010;163(2):238–56. 20608932 10.1111/j.1365-2133.2010.09883.x

[4] Northern Ireland Cancer Registry . Malignant Melanoma. https://www.qub.ac.uk/research‐centres/nicr/CancerInformation/official‐statistics/BySite/MalignantMelanoma/2020. February 10, 2021.

[5] Biswas A , Igali L . Tissue pathway for dermatopathology. London: Royal College of Pathologists; 2019.

